# Clinical Usefulness of Monitoring Muscle Volume during Atezolizumab Plus Bevacizumab Therapy in Patients with Unresectable Hepatocellular Carcinoma

**DOI:** 10.3390/cancers14143551

**Published:** 2022-07-21

**Authors:** Hiroaki Matsumoto, Kaoru Tsuchiya, Hiroyuki Nakanishi, Yuka Hayakawa, Yutaka Yasui, Naoki Uchihara, Keito Suzuki, Yuki Tanaka, Haruka Miyamoto, Shun Ishido, Michiko Yamada, Taisei Keitoku, Tsubasa Nobusawa, Mayu Higuchi, Kenta Takaura, Shohei Tanaka, Chiaki Maeyashiki, Nobuharu Tamaki, Yuka Takahashi, Masayuki Kurosaki, Yasuhiro Asahina, Ryuichi Okamoto, Namiki Izumi

**Affiliations:** 1 Department of Gastroenterology and Hepatology, Musashino Red Cross Hospital, 1-26-1 Kyonancho, Musashino-shi, Tokyo 180-8610, Japan; piroakim530@yahoo.co.jp (H.M.); tsuchiya@musashino.jrc.or.jp (K.T.); nakanisi@musashino.jrc.or.jp (H.N.); yutakay@musashino.jrc.or.jp (Y.Y.); eagle24534n@gmail.com (N.U.); keitosuzuki0106@gmail.com (K.S.); lavi.scarlet@gmail.com (Y.T.); haruketa521@yahoo.co.jp (H.M.); sh.futoball.m@gmail.com (S.I.); p.yamada1992@gmail.com (M.Y.); ktqnet@gmail.com (T.K.); m09201068@gunma-u.ac.jp (T.N.); mayu.h@musashino.jrc.or.jp (M.H.); k.takaura@musashino.jrc.or.jp (K.T.); sheikdrowse@gmail.com (S.T.); c.maeyashiki@musashino.jrc.or.jp (C.M.); nobuharu.tamaki@gmail.com (N.T.); y.takahashi@musashino.jrc.or.jp (Y.T.); kurosaki@musashino.jrc.or.jp (M.K.); 2 Department of Gastroenterology and Hepatology, Tokyo Medical Dental University, 1-5-45 Yushima, Bunkyo-ku, Tokyo 113-8510, Japan; y.hayakawa@musashino.jrc.or.jp (Y.H.); asahina.gast@tmd.ac.jp (Y.A.); rokamoto.gast@tmd.ac.jp (R.O.)

**Keywords:** hepatocellular carcinoma, skeletal muscle, immunotherapy, clinical outcome

## Abstract

**Simple Summary:**

Decrease of skeletal muscle mass index during atezolizumab plus bevacizumab therapy was significantly associated with poor progression-free survival in patients with unresectable hepatocellular carcinoma, while pretreatment sarcopenia was not a significant factor. These findings suggest that monitoring skeletal muscle volume during immunotherapy may be useful for predicting clinical outcomes.

**Abstract:**

Background: Sarcopenia is associated with overall survival in patients with hepatocellular carcinoma (HCC). However, it is not known whether muscle volume is associated with clinical outcomes during combination therapy with immune checkpoint inhibitors. We investigated the relationship between changes in muscle volume and treatment outcomes in patients treated with atezolizumab plus bevacizumab. Methods: Thirty-two patients with HCC who received atezolizumab plus bevacizumab as the first-line treatment between October 2020 and February 2022 were included. Skeletal muscle mass index (SMI) was calculated from the skeletal muscle area at the L3 level of the lumbar vertebrae. We compared pretreatment SMI and SMI at 6–14 weeks after administration. Results: Of the 32 patients, 18 had a decreased SMI, while 14 did not. Progression-free survival (PFS) was significantly longer in patients without SMI decrease than in patients with SMI decrease (8.5 vs. 5.8 months, *p* = 0.011). There were no significant differences in treatment-related adverse events between the patients with and without SMI. Presarcopenia at baseline was not significantly associated with PFS. Conclusions: Decreased SMI was significantly associated with PFS. Monitoring muscle volume during atezolizumab plus bevacizumab therapy is useful in clinical practice.

## 1. Introduction

The incidence of hepatocellular carcinoma (HCC) has been increasing globally and has become the second most common cause of cancer-related deaths [1]. Even though the screening system for HCC in patients with hepatitis B (HBV) or C (HCV) infection has been established in many countries, there is no established HCC surveillance system for patients without a hepatitis viral infection, including non-alcoholic steatohepatitis (NASH) patients. As a result, most patients are diagnosed at intermediate or advanced stages. The BCLC staging system was updated to the 2022 version [2]. In the new BCLC staging system, systemic therapy is strongly recommended in patients with diffuse, infiltrative, or extensive bilobular liver involvement in HCC, even in patients with no extrahepatic metastasis or major vascular invasion. During the era of HCC, systemic therapy has moved to a significant position. According to the results of a phase 3 trial (IMbrave 150) [3], the combination of programmed cell death-ligand 1 (PD-L1) (atezolizumab) and VEGF (bevacizumab) pathway inhibition is believed to be the most effective molecular targeted therapy for advanced HCC and is recommended as the first-line in many countries [4,5]. Previous reports about other molecular targeted agents for HCC, including sorafenib and lenvatinib [6,7], showed that the patients with sarcopenia had poor outcomes. Recently, Fujita et al. [8] reported that overall survival (OS) was significantly lower for the severe psoas mass index (PMI) loss group than for the mild PMI loss group of patients with HCC treated with lenvatinib. In contrast, Akce et al. [9] showed that sex-specific sarcopenia does not predict OS in patients treated with anti-PD-1 antibodies. However, there are no studies on the impact of pretreatment and changes in muscle volume with atezolizumab plus bevacizumab. In this study, we aimed to clarify the clinical impact of pretreatment and changes in muscle volume in patients with HCC who received atezolizumab plus bevacizumab.

## 2. Materials and Methods

### 2.1. Patients

From October 2020 to February 2022, 94 patients with unresectable hepatocellular carcinoma (u-HCC) received atezolizumab plus bevacizumab at the Musashino Red Cross Hospital. Among these patients, those who received atezolizumab plus bevacizumab as first line and had their skeletal muscle mass index (SMI) evaluated before treatment and within 6 to 14 weeks after administration by CT scan were included. To evaluate the association between SMI change and treatment, patients who received less than three courses of atezolizumab plus bevacizumab were excluded. HCC diagnosis was based on the guidelines proposed by the Liver Cancer Study Group of Japan [10], the European Association for the Study of the Liver [11], and the American Association for the Study of Liver Diseases [12]. Treatment decisions were confirmed by the tumor board. The study was conducted according to the Declaration of Helsinki and STROBE guidelines. The study was approved by the Ethics Committee of the Musashino Red Cross Hospital.

### 2.2. Treatment Protocol

Intravenous atezolizumab (1200 mg) plus bevacizumab (15 mg/kg) was administered every three weeks until disease progression or unacceptable toxicity. The treatment was interrupted according to the manufacturer’s guidelines. Dynamic computed tomography was performed at baseline—6–8 weeks after atezolizumab plus bevacizumab administration, and every 6–10 weeks thereafter. Treatment response was evaluated based on the Response Evaluation Criteria in Solid Tumors (RECIST ver1.1). AFP levels were also evaluated at baseline and every 3 weeks thereafter. Adverse events (AEs) were reported according to the Common Terminology Criteria for Adverse Events (CTCAE) version 5.0. To assess changes in liver function, the albumin-bilirubin (ALBI) score [13,14] was calculated at baseline and every 3 weeks.

### 2.3. Image Analysis

Pretreatment CT scans were performed at baseline for tumors within 6 weeks before the administration of atezolizumab and bevacizumab. Initial CT scans were evaluated 6–14 weeks after the administration of atezolizumab and bevacizumab. The skeletal muscle index (SMI) was calculated using the baseline and initial CT images. Transverse images at the L3 level were selected for each scan. The obtained images were analyzed using SliceOmatic software (version 5.0; Tomovision, Montreal, QC, Canada), enabling specific tissue selection using previously determined Hounsfield units (HU). To minimize inter-observer measurement error, we used AVACS, an algorithm that automatically segments skeletal muscle [15]. We evaluated SMI by measuring the muscle mass at the third level of the lumbar spine. We defined presarcopenia as SMI ≤ 42 cm^2^/m^2^ for men and SMI ≤ 38 cm^2^/m^2^ for women according to the JSH guidelines. ΔSMI was calculated using pretreatment SMI and SMI at 6–14 weeks after the administration of atezolizumab and bevacizumab. The median duration of the initial CT scans after the administration was 8 (6–14) weeks. The SMI decrease rate was ΔSMI (SMI at 6 to 14 weeks − pretreatment SMI)/pretreatment SMI × 100 (%). SMI decrease was defined as less than 0.7% decrease using time-dependent ROC analysis. The sensitivity was 0.84, and the specificity was 0.58.

### 2.4. Statistical Analysis

Statistical analyses were performed using EZR version 2.71 (Saitama Medical Center, Jichi Medical University, Saitama, Japan) [16]. Overall survival (OS) was measured from the date of atezolizumab plus bevacizumab administration to the date of death from any cause. Patients who were lost to follow-up were censored at the last date they were known to be alive, and patients who remained alive were censored at the time of data cut-off. PFS was measured as the time from the start of treatment with atezolizumab plus bevacizumab until the date of progression or death from any cause. OS and PFS were analyzed using the Kaplan–Meier method via the log-rank test. Outcome variables were analyzed using the χ^2^ test or Fisher’s exact test for categorical variables, whereas the Mann–Whitney U test was used for continuous variables. A Cox proportional regression hazard model was used to examine the predictors of PFS. Statistical significance was set at *p* < 0.05.

## 3. Results

### 3.1. Overall Efficacy and Safety Data

**Figure 1:**
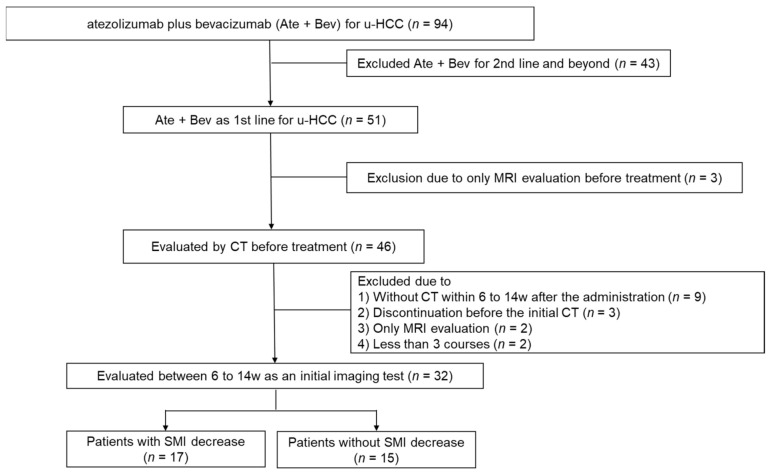
Flow diagram of the study population.

Between October 2020 and February 2022, 94 patients were registered. Of them, 43 received atezolizumab plus bevacizumab as 2nd or later treatment. Five patients were evaluated only by MRI, and 9 patients were not evaluated within 6 to 14 weeks after administration. Three patients discontinued treatment before the initial CT scan, and two patients received less than three courses of atezolizumab plus bevacizumab. A total of 32 patients were included (Figure 1). The baseline patient characteristics are shown in Table 1. At the end of the data cutoff period (30 April 2022), the median duration of follow-up was 10.4 (2.3–17.5) months. The median overall survival (OS) was 10.4 (95% CI, 8.9–11.9) months, and the median PFS was 6.5 (95% CI, 5.5–8.3) months. According to RECIST ver1.1, the radiological evaluation revealed complete response (CR) in 2 patients, partial response (PR) in 8, stable disease (SD) in 15, and progressive disease (PD) in 7. The objective response rate (ORR) and disease control rate (DCR) were 31.3% and 78.1%. Drug discontinuation was reported for 23 patients due to disease progression (*n* = 14) and treatment-related AEs (TRAEs) (*n* = 9).

### 3.2. The Association between SMI Decrease and Treatment Efficacy

**Figure 2:**
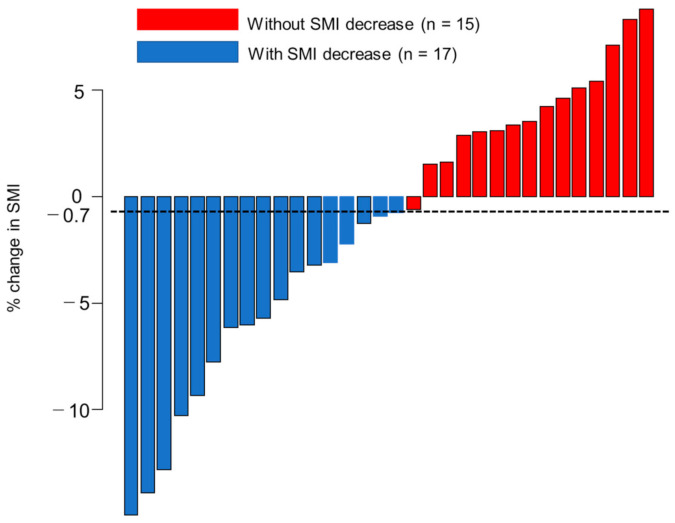
Percent change in SMI during atezolizumab plus bevacizumab.

**Figure 3:**
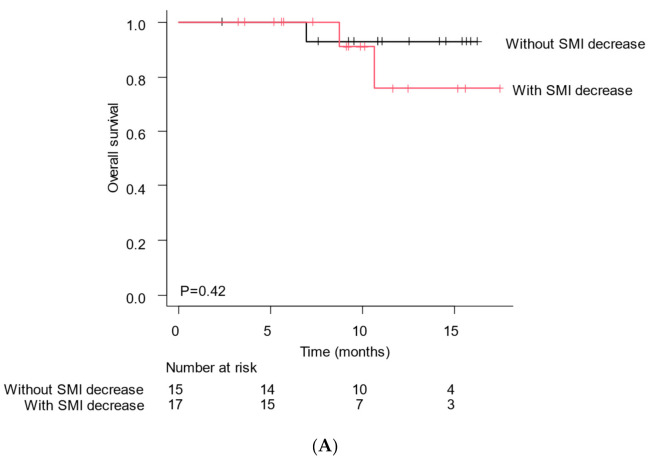

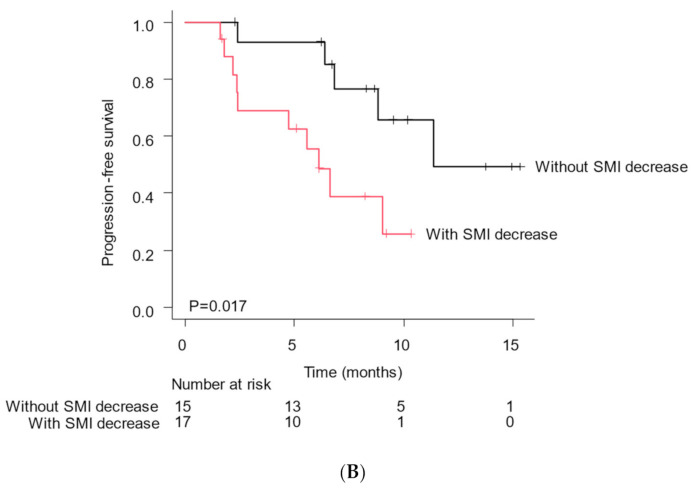
Clinical outcome according to SMI decrease (**A**) Overall survival in patients with or without an SMI decrease. (**B**) Progression-free survival in patients with or without SMI decrease.

Of the 32 patients, 17 showed a decrease in the skeletal muscle mass index (SMI) while 15 did not (Figure 2). A comparison of the baseline characteristics between patients with and without SMI decreases is shown in Table 2. The patients with SMI decrease were significantly older than those without an SMI decrease (with SMI decrease vs. without SMI decrease: 80 vs. 73, *p* = 0.012). However, there were no other differences in the patient background. For the patients with an SMI decrease, the median OS was 9.2 (95% CI, 7.3–11.6) months and PFS was 5.6 (95% CI, 3.8–6.7) months. For the patients without SMI decrease, the median OS was 11.1 (95% CI, 9.3–14.0) months, and PFS was 8.7 (95% CI, 6.6–10.1) months (Figure 3A). The PFS was significantly longer in patients without a decrease (Figure 3B). For the patients without an SMI decrease, complete response (CR) was noted in 2, partial response (PR) in 5, stable disease (SD) in 7, and progressive disease (PD) in 1 patient. For the patients with an SMI decrease, CR was noted in 0, PR in 3, SD in 8, and PD in 6. For the patients without an SMI decrease, the objective response rate (ORR) was 46.7%, and the disease control rate (DCR) was 93.3%. For the patients with an SMI decrease, the ORR was 17.6%, and the DCR was 64.7%. There was no significant difference; however, patients without a decrease in SMI tended to have a better radiological response. (Table 3).

### 3.3. The Association between SMI Decrease and Adverse Events during Atezolizumab Plus Bevacizumab Therapy

Of the 32 patients, adverse events were observed in 15 with hypertension, 10 with proteinuria, 2 with diarrhea, 16 with fatigue, 9 with liver dysfunction, 1 with nausea, and 1 with hypothyroidism. For the 18 patients with an SMI decrease, hypertension was observed in 6 (Grade 1; 2, Grade 2; 4), proteinuria in 5 (Grade 1; 2, Grade 2; 1, Grade 3; 2), diarrhea in 1 (Grade 1; 1), fatigue in 9 (Grade 1; 8, Grade 2; 1), and liver dysfunction in 6 (Grade 1; 5, Grade 2; 1). Nausea or hypothyroidism was not observed. Adverse events were not significantly different from the decrease in SMI. The relative dose intensity (RDI) was 100% for atezolizumab and 96.0% for bevacizumab for 32 patients. In 17 patients with SMI decrease, the RDI until the initial CT was 100% for atezolizumab and 95.1% for bevacizumab. For the 15 patients without a decrease in SMI, the RDI until the initial CT was 100% for atezolizumab and 98.3% for bevacizumab.

### 3.4. The Association between Presarcopenia and Clinical Outcome

Presarcopenia was observed in 14 patients. There were no significant differences in sex, age, or liver function between the patients with and without presarcopenia, while AFP was significantly lower in the presarcopenia group (median AFP 7.7 vs. 363.1, *p* = 0.048). There were no significant differences in PFS between patients with and without presarcopenia (median PFS: 9 months vs. NA: *p* = 0.84). The median OS was not reached in patients with and without presarcopenia.

### 3.5. The Factors Associated with PFS after Administration of Atezolizumab Plus Bevacizumab

We analyzed the factors associated with PFS with atezolizumab plus bevacizumab therapy. Univariate analyses showed that extrahepatic metastasis (yes vs. no: HR 5.4; 95% CI, 1.6–18.9; *p* = 0.0079), SMI decrease (yes vs. no: HR 4.6; 95% CI, 1.3–16.6; *p* = 0.020), and BCLC stage (C vs. B: HR 2.9; 95% CI, 1.0–8.2; *p* = 0.047) were associated with PFS. Multivariate analyses showed that SMI decrease was a significant factor associated with PFS (yes vs. no: HR 5.1; 95% CI, 1.0–21.4; *p* = 0.025) (Table 4).

## 4. Discussion

Since 2020, atezolizumab plus bevacizumab has been recommended as the first-line therapy for unresectable HCC in most countries. To the best of our knowledge, this is the first report to reveal the clinical impact of an SMI decrease with atezolizumab plus bevacizumab. The association between muscle volume and treatment outcomes in patients with metastatic cancer treated with immune checkpoint inhibitors has already been reported by Crombe et al. [17]. According to their results, none of the baseline body composition (BC) parameters correlated with PFS, whereas changes in the psoas muscle area index (PMI) were associated with PFS. In their study, half of the patients had lung cancer, and no patients with HCC were included. Uchikawa et al. [18] showed that patients receiving tyrosine kinase inhibitor (TKI) treatment experienced a significant loss of SMI regardless of disease progression and hepatic reserve. However, there have been no reports on changes in muscle volume during immunotherapy, including atezolizumab plus bevacizumab.

Akce et al. investigated the impact of sarcopenia on the survival of patients treated with an anti-PD-1 antibody. Sarcopenia did not predict OS or PFS in patients treated with the anti-PD-1 antibody. There were no results regarding changes in muscle volume during therapy in their study. Many reports have shown that sarcopenia and presarcopenia are significant factors associated with PFS and OS in patients with advanced HCC treated with TKI, including sorafenib and lenvatinib [19,20,21,22]. In this study, presarcopenia was not significantly associated with PFS. According to the updated results of the phase three trial (IMbrave 150) [23], treatment-related grade three or four adverse events occurred in 43% of patients treated with atezolizumab plus bevacizumab, and 46% of patients received sorafenib. The treatment-related adverse event (TRAE) profile was different between the groups. Proteinuria and hypertension were observed in >20% of patients treated with atezolizumab plus bevacizumab, whereas palmar-plantar erythrodysesthesia syndrome, hypertension, decreased appetite, and diarrhea were reported in >20% of patients treated with sorafenib. Recently, Tada et al. [24] reported the safety and efficacy of atezolizumab plus bevacizumab in older adult patients with HCC. They performed a subgroup analysis of older adult patients aged 75–79, 80–84, or ≥85 years and found no significant differences in OS and PFS among these three groups. They concluded that no significant differences existed in the OS and PFS between the older adults and younger groups. According to previous studies, atezolizumab plus bevacizumab showed better tolerability than tyrosine kinase inhibitors [24,25,26]. Therefore, presarcopenia was not a significant factor associated with PFS with atezolizumab plus bevacizumab therapy.

Our study revealed that the SMI decrease during atezolizumab plus bevacizumab treatment was significantly associated with PFS. The patients with a decrease in SMI were significantly older than those without. Imai et al. [27] reported that age and sex were significantly correlated with a decrease in the L3 SMI in patients with HCC. However, in the multivariate analysis, age was not associated with PFS. Imai et al. [27] also revealed that L3 SMI decreased with increased Child-Pugh score and tumor size in HCC patients. In this study, 14 patients showed a slight increase in SMI after atezolizumab plus bevacizumab treatment. Patients without SMI showed a better radiological response, although the difference was not significant. SMI decrease during atezolizumab plus bevacizumab treatment was the result of complex interactions between patient baseline characteristics (including age, sex differences, etiology, liver function, and comorbidities), aggressive tumor biology, and treatment response. We found that the adverse events and RDI of atezolizumab plus bevacizumab did not differ between patients with and without SMI decrease. Because of the short observation period, the median OS was not reached in patients with and without an SMI decrease. Previous reports have shown that many adipomyokines are secreted by skeletal myocytes or adipocytes, including muscle growth inhibitors (interleukin-6 [IL-6], myostatin, activins A/B, growth, and differentiation factor 15) and muscle growth promoters (follistatin, irisin, bone morphogenetic protein [BMPs], and brain-derived neurotrophic factor) [28]. Myojin et al. [29] recently reported that the PFS and OS of the IL-6-high group were significantly shorter than those of the IL-6-low group of patients with advanced HCC treated with atezolizumab plus bevacizumab. Further investigation into the association between SMI decrease and adipomyokine concentrations is required. For some HCC patients treated with atezolizumab plus bevacizumab, it is difficult to decide on continuation or discontinuation of the therapy based only on imaging findings because there are late responders or pseudo-progression in immunotherapy [30]. The SMI decrease within 6–14 weeks after the administration of atezolizumab plus bevacizumab was significantly associated with PFS and could be useful for developing a treatment strategy for each patient. Even though there are no other studies on SMI decrease during atezolizumab plus bevacizumab therapy for patients with unresectable HCC, patients with SMI decrease would be candidates for BCAA supplementation and should be presented at a multidisciplinary tumor board to discuss additional locoregional therapy or switch to another systemic treatment if the tumor is enlarged.

Our study has some limitations. First, it was a single-center study, and the number of patients was small. Second, there are no data on adipomyokines. Further multicenter investigations, including a larger number of patients, are necessary to establish clinical evidence. Third, the period for CT scans, 6–14 weeks, was wide because of the patients’ situations in real-world practice. Fourth, we could not analyze the effect of BCAA or levocarnitine supplementation because of the few patients who received BCAA and levocarnitine supplementation. BCAA and levocarnitine supplementation is known to prevent the occurrence of sarcopenia in patients with HCC who received systemic therapy [31,32].

This study is the first to reveal the usefulness of monitoring muscle volume during atezolizumab plus bevacizumab therapy using routine CT scans in patients with unresectable HCC. Our results will improve treatment decisions for systemic therapy, especially immunotherapy for HCC.

## 5. Conclusions

SMI decrease was significantly associated with PFS in unresectable HCC patients treated with atezolizumab plus bevacizumab. Monitoring muscle volume during atezolizumab plus bevacizumab would be useful in clinical practice.

## Figures and Tables

**Table 1:** Baseline characteristics of the patients.

Variable	*N* = 32
Sex: Male/Female (%)	Modified ALBI grade: 1/2a/2b/3 (%)
19 (59)/13 (41)	11 (34)/7 (22)/14 (44)/0 (0)
Age (years), median (range)	BCLC stage B/C (%)
77 (54–91)	24 (75)/8 (25)
Height (m^2^), median (range)	Presarcopenia Yes/No (%)
1.6 (1.3–1.9)	14 (44)/18 (56)
Body weight (kg), median (range)	Major vascular invasion Yes/No (%)
59.5 (37.3–87.0)	4 (12)/28 (88)
Etiology HBV/HCV/ALD/NAFLD (%)	Extrahepatic metastasis Yes/No (%)
4 (12)/15 (47)/5 (16)/8 (25)	4 (12)/28 (88)
ECOG PS 0/1/2 (%)	CRP (mg/dL), median (range)
18 (56)/14 (44)/0 (0)	0.25 (0.030–8.8)
Child–Pugh A/B (%)	AFP (ng/mL), median (range)
30 (94)/2 (6)	182.0 (1.60–41,246.2)
ALBI score: median (range)	PIVKAⅡ (AU/mL), median (range)
−2.32 (−3.28∼−1.47)	259.9 (15.0–313,273.7)

Abbreviations: AFP, alpha-fetoprotein; ALBI score, albumin-bilirubin score; BCLC stage, Barcelona Clinic Liver Cancer stage; ECOG, Eastern Cooperative Oncology Group; HBV, hepatitis B virus; HCV, hepatitis C virus; PIVKA-II, protein induced by vitamin K absence or antagonist-II.

**Table 2:** Baseline characteristics of the patients with or without SMI decrease.

Variable	With SMI Decrease (*N* = 17)	Without SMI Decrease (*N* = 15)	*p*
Sex: Male/Female (%)	9 (53)/8 (47)	10 (67)/5 (33)	0.49
Age (years): median (range)	80 (56–91)	73 (54–86)	0.012
Height (m): median (range)	1.6 (1.3–1.8)	1.6 (1.4–1.9)	0.36
Body weight (kg): median (range)	57.2 (40.7–82.5)	60.0 (37.3–87.0)	0.27
Etiology HBV/HCV/ALD/NAFLD (%)	2 (12)/8 (47)/3 (18)/4 (23)	2 (13)/7 (47)/2 (13)/4 (27)	0.70
Child–Pugh A/B (%)	17 (100)/0 (0)	13 (87)/2 (13)	0.21
Pretreatment mALBI score: median (range)	−2.42 (−3.27~−1.71)	−2.23 (−3.28~−1.47)	0.18
Presarcopenia Yes/No (%)	5 (29)/12 (71)	9 (60)/6 (40)	0.15
Discontinuation or reduction of bevacizumab (%)	4 (25)/12 (75)	5 (33)/10 (67)	0.70
Major vascular invasion Yes/No (%)	3 (18)/14 (82)	1 (7)/14 (93)	0.60
Albumin (g/dL), median (range)	3.7 (3.0–4.8)	3.5 (2.8–4.7)	0.29
Total Bilirubin (mg/dL), median (range)	0.7 (0.40–1.6)	0.9 (0.5–1.7)	0.17
CRP (mg/dL), median (range)	0.27 (0.080–2.7)	0.20 (0.030–8.8)	0.21
NH3 (μg/dL), median (range)	35 (12–83)	50 (17–136)	0.05
AFP (ng/mL), median (range)	355.5 (2.2–41,246.2)	21.9 (1.6–4323.6)	0.18
PIVKAⅡ (AU/mL), median (range)	339.2 (15.4–313,273.7)	144.5 (15.0–13,385.4)	0.84

Abbreviations: AFP, alpha-fetoprotein; ALD, alcoholic liver disease; HBV, hepatitis B virus; HCV, hepatitis C virus; mALBI score, modified albumin-bilirubin score; NAFLD, non-alcoholic fatty liver disease; PIVKA-II, protein induced by vitamin K absence or antagonist-II; SMI, skeletal muscle mass index.

**Table 3:** Radiological evaluation.

Variable	With SMI Decrease (*N* = 17)	Without SMI Decrease (*N* = 15)	*p*
CR	0	2	
PR	3	5	
SD	8	7	
PD	6	1	
ORR	17.6%	46.7%	0.13
DCR	64.7%	93.3%	0.088

Abbreviations: CR, complete response; DCR, disease control rate; ORR, objective response rate; PD, progressive disease; PR, partial response; SD, stable disease; SMI, skeletal muscle mass index.

**Table 4:** Univariate and multivariate of PFS.

Variable	Univariate Analysis			Multivariate Analysis		
	HR	95% CI	*p*	HR	95% CI	*p*
sex	0.95	0.34–2.68	0.92			
Age (years)	1.0	0.94–1.1	0.80			
ALD	1.8	0.49–6.5	0.38			
Child-Pugh score	0.76	0.33–1.7	0.51			
Major vascular invasion	0.48	0.063–3.7	0.48			
Extrahepatic metastasis	5.4	1.56–18.9	0.0079	1.8	0.26–12.1	0.57
Presarcopenia	1.1	0.40–3.1	0.84			
SMI decrease	4.6	1.3–16.6	0.020	5.1	1.0–21.4	0.025
BCLC stage C	2.9	1.0–8.2	0.047	1.3	0.28–6.4	0.72
Baseline CRP (mg/dL)	1.3	1.0–1.6	0.033	1.4	1.0–1.9	0.052
Baseline AFP ≥ 400 (ng/mL)	1.4	0.42–4.3	0.61			

Abbreviations: AFP, alpha-fetoprotein; ALD, alcoholic liver disease; BCLC stage, Barcelona Clinic Liver Cancer stage; HR, hazard ratio; PFS, Progression-free survival; SMI, skeletal muscle mass index; 95% CI, 95% Confidence Interval.

## Data Availability

The data presented in this study are available upon request from the corresponding author.
